# Tribological Behavior of the Laser Micro-Textured PEEK-1040 Steel Friction Pairs

**DOI:** 10.3390/polym17050645

**Published:** 2025-02-27

**Authors:** Risheng Long, Haiming Wang, Jincheng Hou, Qingyu Shang, Yimin Zhang, Lin Zong, Zhijun Zhang

**Affiliations:** 1 Equipment Reliability Institute, Shenyang University of Chemical Technology, Shenyang 110142, China; hjcjc312@163.com (J.H.); sqycan@163.com (Q.S.); zhangyimin@syuct.edu.cn (Y.Z.); 2 Liaoning Provincial Key Laboratory of Efficient Chemical Mixing Technology, Shenyang 110142, China; 3 School of Mechanical and Power Engineering, Shenyang University of Chemical Technology, Shenyang 110142, China; 15524087957@163.com; 4 School of Mechanical Engineering and Automation, Northeastern University, Shenyang 110819, China; zhjzhang@mail.neu.edu.cn

**Keywords:** PEEK-1040 steel friction pair, surface micro-texturing, tribological performance, full-film lubrication

## Abstract

To compare them with PTFE-40# steel tribo-pairs, the tribological properties of textured PEEK-40# (AISI 1040) steel friction pairs were researched under full-film lubrication conditions by manufacturing micro-dimples with different dimensions on the contact surfaces of 1040 steel discs using laser surface texturing (LST). After repeated tribological tests, the coefficients of friction (COFs), wear losses, and wear morphologies of the PEEK-1040 steel friction pairs were measured and analyzed. The results show that micro-dimples do not significantly reduce the average COFs of PEEK-1040 steel friction pairs when lubricated with a sufficient amount of hydraulic oil, but they do reduce the wear losses of most groups. When the dimple diameter was 250 μm, the dimple depth was 5 μm, the area ratio was 6.6%, and the mass loss of the 1040 steel disc was reduced by 90% compared to the smooth reference. In comparison to the behavior of the PTFE-1040 steel tribo-pairs, PEEK-1040 steel friction pairs can provide better tribological performance, whether smooth or dimple-textured. This study offers important insights for the design of seals in machinery.

## 1. Introduction

The rectangular wear-resistant slider and O-ring are the main components of Glay rings, which are typically used to realize dynamic sealing in hydraulic cylinders [1]. The wear-resistant slider is typically manufactured using self-lubricating materials, e.g., polytetrafluoroethylene (PTFE), polyoxymethylene (POM), and polyether-ether-ketone (PEEK) [2,3]. Severe wear of the rectangular slider and the inner wall is ineluctable and a primary cause of hydraulic cylinder failure [4,5]. Enhancing the tribological performance of these contact surfaces is crucial for extending the service life and reducing the energy consumption of hydraulic cylinders.

Structural optimization [6], the development of new materials, and making modifications to current materials [7] are effective approaches to reducing wear in sealing components. Similar to PTFE [8], PEEK is also widely used in mechanical parts due to its excellent resistance to temperature, wear, fatigue, and aging [9,10,11]. Since it was first proposed in 1966 [12], surface texturing (ST) technology has been proven to be a highly accurate [13,14], relatively fast [15,16], low-cost [17,18,19], and environmentally friendly method [20] for improving the tribological performance of mechanical parts [21,22,23], under various lubrication conditions [17,18,24,25,26], by manufacturing micro-textures [27,28,29,30] with appropriate parameters [31,32,33] on the contact surfaces. In relation to hydraulic cylinders, laser surface texturing (LST) has been researched to tune the tribological behavior of seals, such as silicon carbide sealing rings [34], non-contacting mechanical face seals [35], and two-phase mechanical face seals [36].

Currently, research on the tribological behavior of micro-textured polymer–steel friction pairs is quite limited. Similar to the PTFE-40# steel tribo-pairs analyzed in previous work [37], in this study, according to the orthogonal experimental method, micro-dimples, with three parameters (i.e., dimple diameter, dimple depth, and area ratio) varied at three levels, were fabricated on 40# steel (AISI 1040) discs using a laser-marking machine and then tested against PEEK rings under full-film-lubrication conditions. The results will serve as valuable references for the design of commonly used seals in mechanical devices.

## 2. Materials and Methods

### 2.1. Materials and Pretreatment

**Figure 1:**
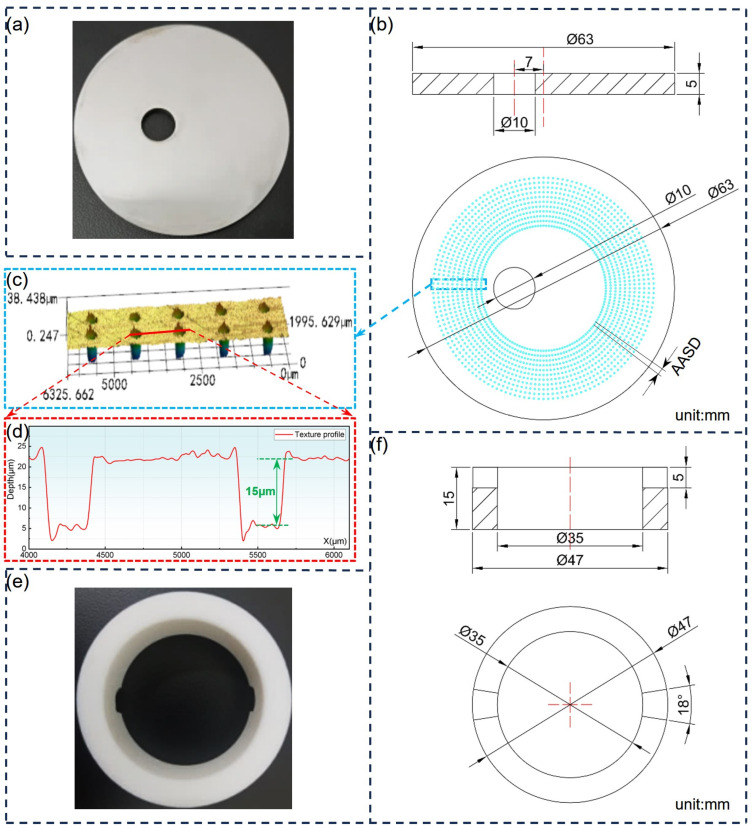
(**a**) Photo of the 1040 steel disc; (**b**) section view and texture design of the 1040 steel disc; (**c**) representative 3D morphology of dimple-textured surface of the 1040 steel disc before being re-polished; (**d**) sectional profile curve of micro-dimples in (**c**); (**e**) photo of PEEK ring; (**f**) section and top views of the PEEK ring.

The dimensions of the 1040 steel disc and PEEK ring are listed in Table 1. The surface hardness of the 1040 steel disc was HB114. As shown in Figure 1, a Φ10 mm hole with an eccentricity of 7 mm was laser-cut directly in each disc to prevent the disc from rotating during the tribo-test. Prior to each test, the contact surfaces of the steel disc and PEEK ring were polished to Ra 0.8 μm and Ra 1.1 μm, respectively, using sandpaper of various degrees.

Note that due to its excellent self-lubricating and mechanical properties compared to PTFE, PEEK was chosen in this work to research its tribological performance as tested with 1040 steel discs under the same experimental conditions as in ref. [37].

A fiber laser-marking machine (WQ-30W, Wanquan, Shenyang, China) was used to manufacture micro-dimples on the surfaces of the 1040 steel discs, which had been pre-washed in acetone solution in an ultrasonic tank in advance.

### 2.2. Design of Experiment and Laser-Marking Process

Based on previous works [19,25,37,38], three factors (dimple diameter (*D*), dimple depth (*H*), and area ratio (*P*)) were chosen and varied at three levels (1, 0, and −1, see Table 1) according to the orthogonal design method (ODM). In ODM, orthogonal tables are used to select a representative set of “uniformly distributed and comparable” points through a comprehensive experiment. This allows for significantly fewer tests while still providing a clear explanation of the relationship between experimental conditions and performance indicators (see Supplementary Materials). A total of 17 groups were designed and auto-named using Design Expert 10 (a widely used commercial software product for displaying important elements and optimizing the settings of a process to achieve high performance). The dimple parameters in X5-1~X5-5 were kept consistent to verify the reproducibility of the results and the fitting of the prediction model.

To ensure that the area ratio was accurately maintained at three levels, 144/240 (only T2) sets of uniformly radiating distributed micro-dimples were prepared around the circumference of each textured group; i.e., the angle between two adjacent sets of dimples (AASD) was 1.5°/2.5° (see Figure 1b). Each set had different micro-dimples, and the total quantities of dimples for each group are listed in Table 1. A smooth reference was labeled as CT. In addition, the total effective volume of dimples (TEVD) was defined as TEVD = ((3.14 × *D*^2^)/4) × *H* × *T* × 10^−9^ to reflect the maximum collection and storage capacity of the textured surfaces. The actual dimensions of micro-dimples were within the following ranges: 200 ± 1.8 μm; 250 + 2.4 μm; 300 ± 3.2 μm; 5–7.2 μm; 15–17.3 μm; and 25–26.4 μm.

### 2.3. Tribological Test and Characterization

A vertical universal tribo-test rig (MMW-1A, Huaxing, Jinan, China) was used to test the PEEK-1040 steel friction pairs at room temperature (18 ± 2 °C), as they were completely submerged in hydraulic oil (L-HM32, Kunlun, China), using a ring-on-disc setup [37]. Considering the typical working pressure (≤25 MPa) and speed (≤3 m/s) of key seals in hydraulic cylinders, as well as the operational limits of the tribo-test rig, the vertical load was set to 1000 N, and the rotating speed was set to 200 revolutions per minute (RPM). Each test lasted 2400 s, corresponding to a sliding distance of approximately 1 km.

The melt bulges in Figure 1c,d formed during the laser-marking process should first be removed using sandpaper before the tribo-test. After mounting the samples, a sufficient amount of hydraulic oil (25 mL) was poured into the lower fixture. The coefficient of friction (COF) curves were measured and recorded directly using the rig. A high-precision (0.1 mg) electronic analytical balance (EX225D, Ohaus, Parsippany, NJ, USA) was used to measure the mass losses of the samples.

Groups X5-1~X5-5 were only tested once due to their identical parameters. The other groups were repeatedly tested using three new 1040 steel discs to guarantee the data were accurate and reduce the effects of random factors. Several PEEK rings were reused after being re-polished for the small wear amount observed on them in each tribo-test. Finally, the worn surfaces were characterized using a 3D surface profilometer (VK-X1050, Keyence, Osaka, Japan). To wholly characterize the entire contact surface (approximately 6 mm), all measurements were taken at a magnification of 200× (i.e., eyepiece, 20×; objective lens, 10×), and then the data were pieced together using the software product included with the profilometer.

## 3. Results and Discussion

### 3.1. Coefficients of Friction

**Figure 2:**
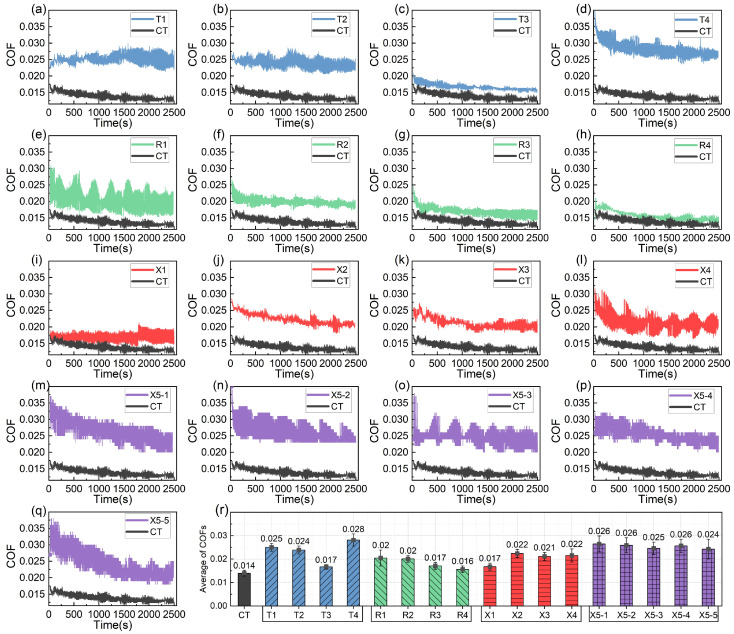
COF data of the PEEK-1040 steel friction pairs under full-film lubrication conditions: (**a**–**d**) COF curves of T1-T4; (**e**–**h**) COF curves of R1-R4; (**i**–**l**) COF curves of X1–X4; (**m**–**q**) COF curves of X5-1~X5-5; (**r**) average COFs of 17 groups.

The COF curves of the different groups, obtained as the PEEK rings were tested against the dimple-textured 1040 steel discs, are shown in Figure 2. The COF curve of the CT group was added as a reference. Initially, all COF curves show high values. From the 200th onwards, the curves begin to decline and then gradually stabilize. The CT group exhibited the lowest COF curve, with only slight fluctuations. In contrast, the COF curves of the micro-textured groups were relatively high throughout the tests, and most of them showed more intense fluctuations (see Figure 2a–q). As a result, their average COFs were all larger than the COF of the smooth reference. The average COF (0.016) of R4 was the lowest but still higher than the value of 0.014 observed in the CT group (see Figure 2r). Therefore, micro-dimples did not improve the friction-reducing behavior of the PEEK-1040 steel friction pairs under the conditions used in this work.

The obvious periodic fluctuations in the COF curves are likely due to the sticky sliding or “crawling” phenomena between the PEEK rings and the 1040 steel discs. This is because the static COF between PEEK and steel is approximately 0.3–0.4, much higher than the average COF (0.014–0.028) observed in this work. When the TEVD ranged between 0.8 and 1.2 (T3 and X2), the fluctuations in the COF curves tended to be significantly reduced. Among the 17 groups, the COF curves of T3 (D200-P10.75-H5), R3 (D300-P10.75-H5), R4 (D300-P10.75-H25), and X1 (D250-P6.6-H5) are notably lower than those of the other groups.

For the groups where the dimple diameter was 200 μm (T1–T4), the COF curves were much higher than that of the CT group (see Figure 2a–d). Conversely, for the groups with a dimple diameter of 300 μm (R1–R4), the COF curves were much closer to that of the smooth reference (see Figure 2e–h), with their average COFs being significantly lower than those of the other textured groups. When the dimple diameter was 250 μm (X1–X4), the COF curves and average COFs mostly fell between those of T1–T4 and R1-R4 (see Figure 2i–l). Notably, when the dimple depth was shallow (5 μm) and the area ratio was not too large (6.6% and 10.75%), the COF curves tended to be lower, as seen in T3, R3, and X1. The COF curves of X5-1~X5-5 were similar (see Figure 2m–q), with intense fluctuations, and their average COFs were also evidently higher than the COF of the smooth group.

### 3.2. Wear Morphologies and Mass Losses

**Figure 3:**
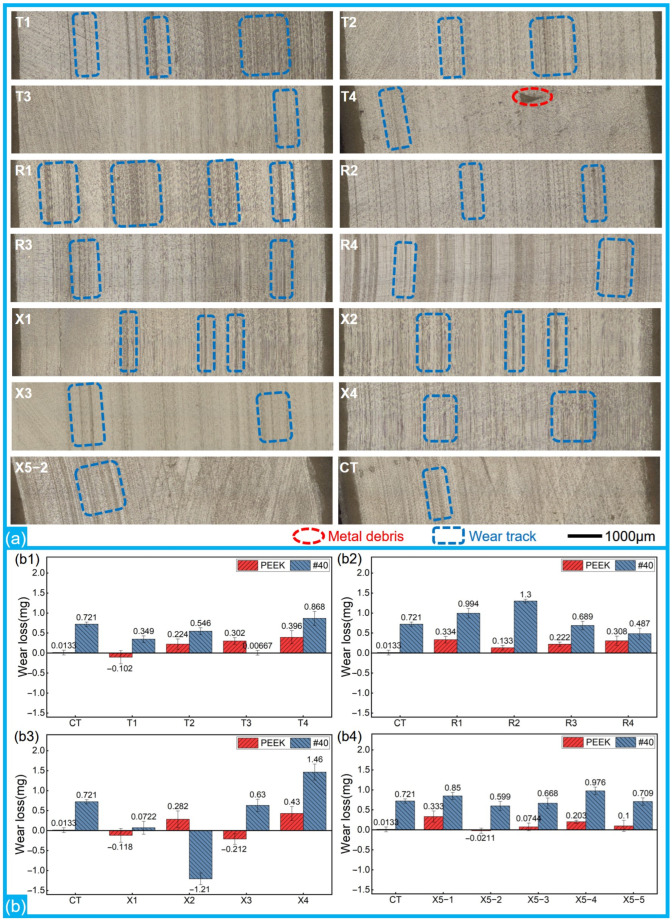
Representative wear morphologies and wear losses: (**a**) wear morphologies of the PEEK rings after ultrasonic washing; (**b**) wear losses of the PEEK rings and 1040 steel discs: (**b1**) wear losses of T1–T4; (**b2**) wear losses of R1–R4; (**b3**) wear losses of X1–X4; (**b4**) wear losses of X5-1~X5-5.

**Figure 4:**
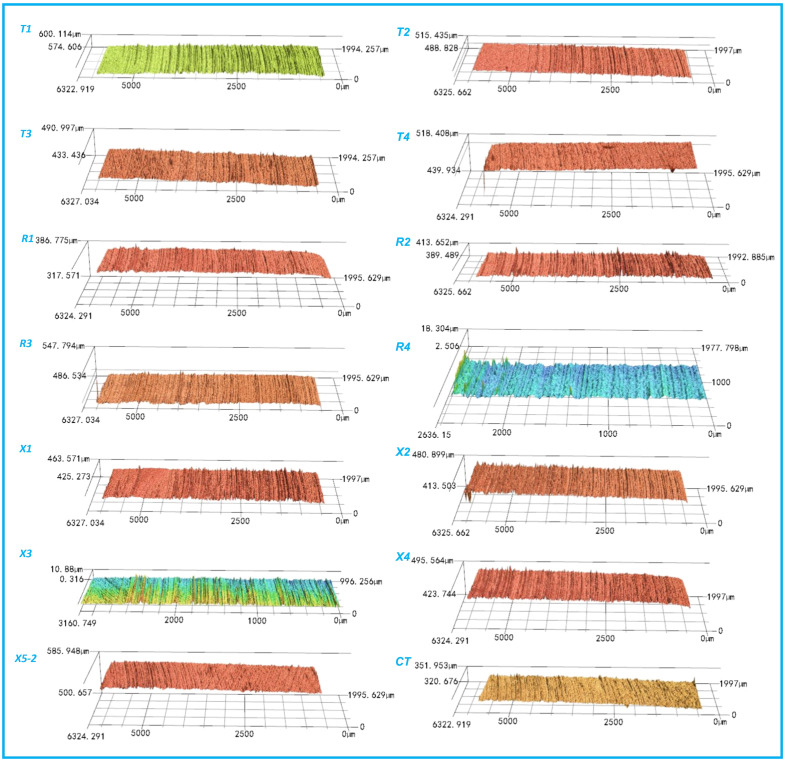
Representative three-dimensional wear morphologies of the PEEK rings after ultrasonic washing.

Figure 3 shows the representative wear morphologies of the PEEK rings and the mass losses of different friction pairs. There were wear marks, whether slight or severe, on the surfaces of both the smooth and textured groups. Overall, compared to the CT group, there were more and deeper wear marks on the surfaces of textured groups, especially R2 and X4. The marks on the PEEK rings in T3, T4, R3, and X3 were few and shallow, as confirmed by their 3D morphologies in Figure 4 (enlarged 2000% in the height direction).

Despite their awful friction-reducing performance, the mass losses of most of the micro-textured groups were lower than the losses of the smooth reference, suggesting good anti-wear performance of the PEEK-1040 steel friction pairs. When the dimple diameters were 200 μm and 250 μm, the wear resistances of the textured PEEK-1040 steel friction pairs were noticeably enhanced, except in groups with a great dimple depth (25 μm, T4 and X4). When the dimple diameter was 300 μm, the mass losses of R1 and R2 were significantly larger than those of the CT group for both the PEEK rings and the 1040 steel discs. The losses of R4 were the lowest among the R1-R4 groups and better than those of the CT group. Among the 17 groups, X1 (D250-P6.6-H5) exhibited the best anti-wear properties. Some PEEK rings (T1, X1, and X3) showed an increase in mass after ultrasonic washing due to the embedded metal debris on the contact surfaces. Compared to the PTFE rings in ref. [37], the higher hardness of PEEK resulted in less metal debris being embedded on the worn surfaces of the PEEK rings. The wear amounts of the PEEK rings in groups CT, T3, X5-2, and X5-5, whether positive or negative, were lower than the accuracy of the balance and negligible.

**Figure 5:**
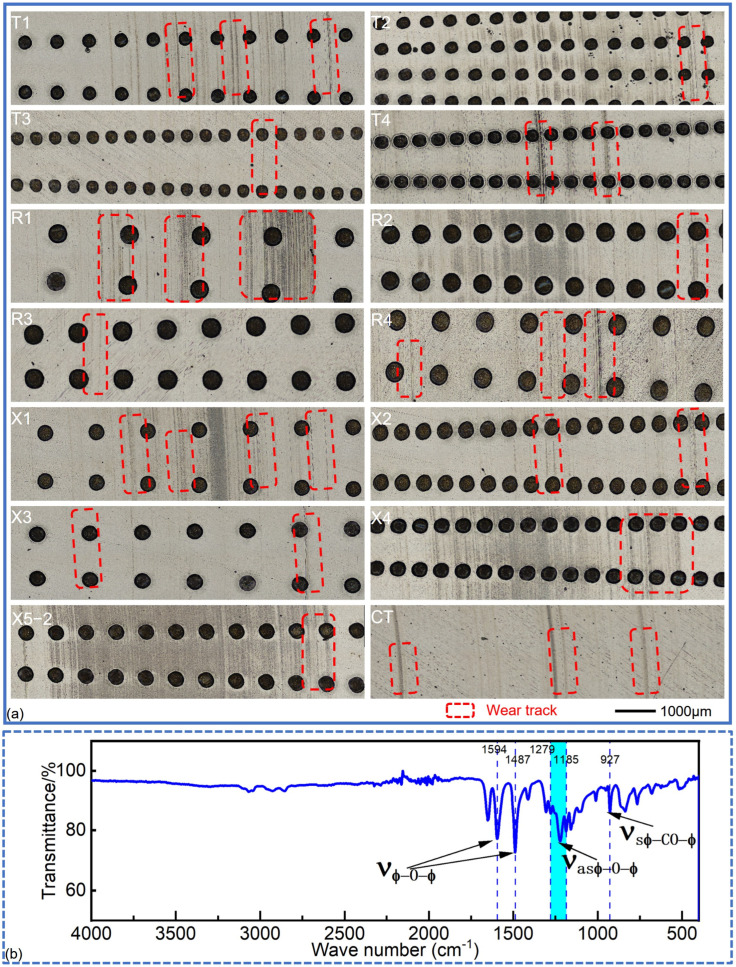
Representative wear morphologies of the 1040 steel discs after ultrasonic washing and the characteristic infrared absorption peaks of the PEEK transfer film: (**a**) wear morphologies of the 1040 steel discs; (**b**) typical infrared spectral characteristics of the PEEK collected on the surface of 1040 steel discs.

**Figure 6:**
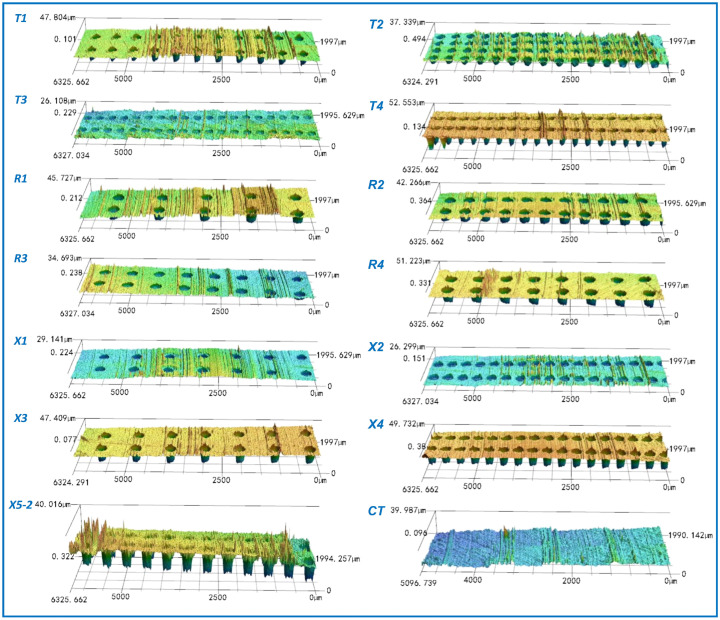
Representative three-dimensional wear morphologies of the 1040 steel discs after ultrasonic washing.

Figure 5 shows the representative wear morphologies of the 1040 steel discs and the characteristic infrared absorption peaks observed. There were several deep wear marks on the contact surfaces of the smooth group. Similarly, fewer marks were seen on the T3 (D200-P10.75-H5), R3 (D300-P10.75-H5), and X3 (D250-P6.6-H25) groups. This suggests that a shallower depth of dimples (5 μm) can reduce wear marks on the surfaces, which is consistent with the relatively smaller average COFs and mass losses of these groups. When the dimple depth was 15 μm, more wear marks appeared on the surfaces of the 1040 steel discs, regardless of the area ratios. When the dimple depth was 25 μm, only a few prominent wear marks were visible on the steel discs, as the area ratios were relatively small, e.g., for X3 (D250-P6.6-H25), T4 (D200-P10.75-H25), and R4 (D 200-P10.75-H25). However, when the area ratio was 14.9%, more wear marks appeared on the steel discs, as observed for the X4 (D250-P14.9-H25) group. Furthermore, for the dimple-textured groups that showed more extensive wear marks on the contact surface (i.e., T2 (D200 -P14.9-H15), R1 (D200-P14.9-H15), R2 (D300-P14.9-H5), X1 (D250-P6.6-H5), X4 (D250-P14.9-H25), and X5-2 (D250-P10.75 -H15)), more intense fluctuations were observed in their COF curves. This also suggests that there was a ’crawling’ phenomenon between the PEEK rings and 1040 steel discs. It should be noted that some of the transfer film on the 1040 steel discs could not be completely removed after ultrasonic washing. Correspondingly, as shown in Figure 6 (enlarged 2000% in the height direction), 5–10 μm high micro-peaks were present on the surfaces of the 1040 steel discs. This explains the negative mass loss of the 1040 steel disc in X2 (see Figure 3(b3)), and it also implies that the calculated mass losses of the steel discs are slightly smaller than the actual values. Additionally, cavitation on the 1040 steel discs was not significant, unlike what was observed in PTFE-40# steel tribo-pairs [37,39].

The transfer film collected from the wear steel discs was analyzed using Fourier transform infrared spectroscopy (FTIR, IS10, Thermo Fisher Scientific, Waltham, MA, USA). The characteristic infrared absorption peaks of the main functional groups of PEEK were observed at wave numbers of 1549 cm^−1^ and 1487 cm^−1^, corresponding to the Ar-O-Ar plane vibration mode in PEEK molecules (ν_Φ-O-Φ_), as shown in Figure 5b [40]. Another absorption peak appeared in the PEEK spectrum in the range of 1279~1185 cm^−1^, corresponding to the asymmetric stretching vibration mode of Ar-O-Ar in PEEK (ν_asΦ-O-Φ_).

### 3.3. Discussion

**Figure 7:**
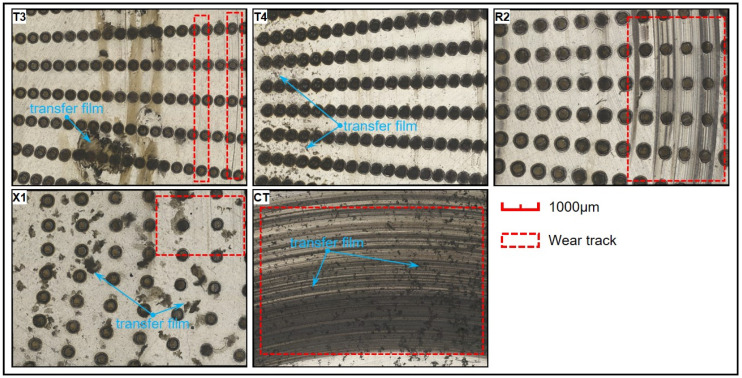
Representative wear morphologies of the 1040 steel discs with PEEK transfer films before ultrasonic washing.

The influences of the micro-dimples on the tribological behavior of PEEK-1040 steel friction pairs, fully immersed in anti-wear hydraulic oil, can be summarized as follows:

(1) When there is a sufficient amount of hydraulic oil, the micro-dimples fill with oil, and a lubricating oil film gradually forms on the contact surfaces of the friction pair. In this case, the average COF of the smooth PEEK-1040 steel friction pair was quite low, with minimal fluctuations in its COF curve. In contrast, in the dimple-textured groups, hydraulic oil in micro-dimples could be squeezed out and replenished due to the combined effect of centrifugal force and the continuous pressing of the PEEK ring [37,41]. Through acting like numerous “micro-hydrodynamic bearings”, the micro-dimples can greatly slow down lubricant migration and improve the load-carrying capacity (LCC) of the oil film [37,39]. Despite the self-lubricating properties of PEEK, its relatively high surface hardness and the greater surface roughness of the textured surface still lead to severe wear on both the PEEK rings and the steel discs. This explains why dimples do not reduce the average COFs of the PEEK-1040 steel friction pairs under the conditions used in this work.
(2) The static COF between PEEK and steel surface is around 0.3–0.4, which is much higher than the average COF (0.014–0.028) observed in this work. This is the primary cause of the “crawling” or “creeping” phenomena in PEEK-1040 steel friction pairs, resulting in large and obvious fluctuations in their COF curves. Compared to the smooth reference, some textured groups exhibited more-severe COF fluctuations and more significant wear marks on their contact surfaces, which also led to their higher average COFs. In addition, an appropriate TEVD (0.5–0.9) reduced the average COFs of the micro-textured groups in this work.
(3) During the continuous radial migration of the lubricant, micro-dimples can effectively trap and store particles or debris carried by oil, reducing the amount of rigid metal debris left on the contact surfaces. Given the relatively high surface hardness of PEEK, only a small amount of metal debris was embedded in the contact zones of the PEEK rings. This explains the negative mass losses of some of the PEEK rings (T1, X1, and X3 in Figure 3b), notably different from the morphologies observed for PTFE rings in ref. [37]. Additionally, discontinuous PEEK transfer films form on the steel disc (see Figure 7) [18,19]. These transfer films directly affect the tribological properties of PEEK-1040 steel friction pairs, whether smooth or dimple-textured. Due to the higher surface energy of PEEK, the bond between PEEK transfer films and the steel discs is relatively strong, particularly under the continuous compression of the PEEK rings. This explains why some PEEK residues stick on the steel discs after ultrasonic washing. Regarding the textured groups, due to the collection and storage of wear debris in the micro-dimples, fewer and more uneven-distributed PEEK transfer films remain on the contact surfaces, contributing to their higher average COFs, especially when the dimple diameter is relatively small (T1–T4, X1–X4, and X5-1~X5-5). When the dimple diameter is larger (300 μm), the presence of a residual transfer film on the surface is almost invisible, which explains the lower average COFs and COF curves for these groups.

(4) A high area ratio corresponds to a greatly reduced contact area. In this work, as the area ratio remained constant, an increase in dimple diameter resulted in fewer micro-dimples (see Table 1) and a great distance between adjacent micro-dimples. Therefore, when the area ratio was relatively small (6.6%), more PEEK transfer films and debris remained on the contact surfaces, and the degree of improvement in the load-bearing capacity due to the micro-eddies in the dimples was limited. This led to a deterioration tribological behavior of the PEEK-1040 steel friction pairs, especially when the dimple diameter was 300 μm, which is the primary reason for the poor tribological performance of R1 (D300-P6.6-H5). A moderate area ratio (10.75%) and smaller dimple depth (5 μm) tend to reduce average COFs, COF fluctuations, and wear losses (T3 and R3). This is because the transfer films on the contact surfaces are more uniform, and the micro-eddies in the dimples are more effective in this case. As the area ratio is large (14.9%), fewer PEEK transfer films and less debris remain on the contact surfaces, and the load-bearing capacity is improved by the micro-eddies in the dimples too. As a result, the tribological properties of the PEEK-1040 steel friction pairs improve, particularly when the dimple depth is 5 μm, as seen in X2, although the surface roughness of the contact surfaces increases. The good tribological performance of X1 can be attributed to its greater number of micro-dimples (compared to R1) and its shallower dimple depth.
(5) When the dimple depth is greater (15 and 25 μm), the transfer films become more uneven, and the efficiency of the “micro-hydrodynamic bearings” decreases for the “self-sealing” effect [18,19], especially when the dimple depth is 25 μm. As a result, the average COFs and the fluctuation in COF curves worsen, particularly when the diameter of the dimple is relatively small (T1–T2, T4, and X3–X4). This also explains the relatively higher mass losses for this group. The good tribological performance of R4 can mainly be attributed to its large dimple diameter (300 μm) and moderate area ratio (10.75%). In addition, the formation of “U-shaped thin-wall embedded units” [42] and laser-induced phase transitions may also refine the intrinsic material structure, increasing the surface-hardness and enhancing the anti-wear performance of micro-textured discs [18].

Compared with the data on PTFE-1040 tribo-pairs reported in previous work [37], the average COFs and wear losses of the PEEK-1040 steel friction pairs were significantly reduced under the same conditions. The smooth PEEK-1040 steel friction pair already provided excellent overall tribological performance, with only minor fluctuations in its COF curve. There was a large error in the prediction model obtained using the response surface methodology (RSM) (see Supplementary Materials). Although the average COF (0.018) of the optimized PEEK-1040 steel friction pair is higher than the lowest value in Figure 2r, it is still much lower than that (0.024) of the optimized PTFE-1040 steel tribo-pair in ref. [37]. Furthermore, the wear amounts of PEEK rings and 1040 steel discs after optimization were only 0.025 mg and 0.045 mg, respectively, both of which are significantly lower than the wear losses of the PTFE ring and 40# steel disc in the optimized PTFE-1040 steel tribo-pair (0.34 mg and 0.27 mg).

## 4. Conclusions

(1) Micro-dimples did not effectively reduce the average COFs or the COF fluctuations of the PEEK-1040 steel friction pairs under full-film-lubrication conditions. Despite their poor friction-reducing properties, the mass losses in most of the dimple-textured groups were lower than those in the CT group, suggesting that micro-dimples could enhance the anti-wear performance of the PEEK-1040 steel friction pairs in this case. Among the 17 groups, X1 exhibited the best anti-wear properties.
(2) When the dimple diameter is 300 μm, the COF curves of R1–R4 are much closer to that of the smooth reference, and their average COFs are significantly lower than those of the other textured groups. When the area ratio is moderate (10.75%) and the dimple depth is shallow (5 μm), as seen in T3, the dimple-textured PEEK-1040 steel friction pair demonstrates acceptable tribological properties. Its average COF (0.017) is only 0.003 higher than that of the CT group, while its mass loss for the 1040 steel disc is much lower than the loss (0.87 mg) of the smooth reference.
(3) The average COFs and mass losses of the PEEK-1040 steel friction pairs were lower than those of the PTFE-1040 steel tribo-pairs under the same conditions. The smooth PEEK-1040 steel friction pair demonstrated excellent overall tribological performance, with only small fluctuations in its COF curve. Parameter optimization could not effectively reduce the average COF of the PEEK-1040 steel friction pair, but the wear amounts of PEEK rings and 1040 steel discs after optimization were only 0.025 mg and 0.045 mg, respectively, both of which are significantly lower than the mass losses of the PTFE ring and 1040 steel disc in the optimized PTFE-1040 steel tribo-pair.

Exploring new polymer materials for wear-resistant rings used in hydraulic cylinders, and understanding their synergistic anti-wear and friction-reduction behavior for micro-textured surfaces, is an area that requires ongoing and in-depth research.

## Figures and Tables

**Table 1:** Dimensions of friction pairs, factor-level table, and groups designed using Design Expert 10.

1040 Steel Disc				PEEK Ring			
Diameter, mm		63		Outer diameter, mm		47	
Thickness, mm		5		Inner diameter, mm		35	
				Thickness, mm		15	
**Factor**		**Level**					
		−1		0		1	
Dimple diameter/*D*, μm		200		250		300	
Dimple depth/*H*, μm		5		15		25	
Area ratio/*P*, %		6.6		10.75		14.9	
**Group ID**	***D*/μm**	***P*/%**	***H*/μm**	**Sets**	**Dimples of one set**	**Total Dimples, *T***	TEVD *
T1	200	6.6	15	144	24	3456	1.628
T2	200	14.9	15	240	32	7680	3.617
T3	200	10.75	5	144	39	5616	0.882
T4	200	10.75	25	144	39	5616	4.409
R1	300	6.6	15	144	11	1584	1.679
R2	300	14.9	15	144	24	3456	3.662
R3	300	10.75	5	144	17	2448	0.865
R4	300	10.75	25	144	17	2448	4.324
X1	250	6.6	5	144	15	2160	0.530
X2	250	14.9	5	144	34	4896	1.201
X3	250	6.6	25	144	15	2160	2.649
X4	250	14.9	25	144	34	4896	6.005
X5-1	250	10.75	15	144	25	3600	2.649
X5-2	250	10.75	15	144	25	3600	2.649
X5-3	250	10.75	15	144	25	3600	2.649
X5-4	250	10.75	15	144	25	3600	2.649
X5-5	250	10.75	15	144	25	3600	2.649

*: TEVD: total effective volume of dimples.

## Data Availability

The raw data supporting the conclusions of this article will be made available by the authors on request.

## Supplementary Materials

The following supporting information can be downloaded at: https://www.mdpi.com/article/10.3390/polym17050645/s1, Table S1: Average COFs input for the BBD-RSM model; Table S2: Analysis of the BBD-RSM model; Figure S1: Response surfaces of the interaction among three factors (*D*, *P*, *H*) on the average COFs of the PEEK-1040 steel friction-pairs. (a) Response surface between *P* and *H*; (b) Response surface between *D* and *P*; (c) Response surface between *D* and *H*; Figure S2: The COF curves and wear losses of three groups (OT, LT and CT). (a) COF curves of OT, LT and CT; (b) wear losses of OT, LT and CT; Figure S3: Representative worn surfaces and morphologies of the contact-surfaces of the OT, LT and CT groups after ultrasonic cleaning. (a) PEEK rings; (b) 1040 steel discs.
