# Supplementary material for: Tribological Behavior of the Laser Micro-Textured PEEK-1040 Steel Friction Pairs
Source: Polymers (Basel). 2025 Feb 27;17(5):645. doi: 10.3390/polym17050645 (PMC11902717; doi:10.3390/polym17050645)
Supplement: Supplementary file 1 [file polymers-17-00645-s001.zip › polymers-3467785-supplementary.pdf]

Parameter optimization and experimental validation

Risheng Long [1, 2*], Haiming Wang [3], Jincheng Hou [1], Qingyu Shang [1], Yimin Zhang [1], Lin Zong [3 *], Zhijun Zhang [4]

1 Parameter optimization by BBD-RSM

The COF data of the 17 groups used for the BBD-RSM analysis are listed in Table S1. Each average COF presents the average value for the entire COF curve of the corresponding group. Evidently, the average COFs of PEEK-1040 steel friction-pairs are quite lower than those of PTFE-1040 steel tribo-pairs under the same conditions [37]. For instance, the average COF of smooth group is only 0.01398, much lower than that (0.0397) of smooth group in Ref. [37]. The analyses of the derived BBD-RSM model are shown in Table S2.

Table S1. Average COFs input for the BBD-RSM model.

Group name	Average COF
T1	0.02506
T2	0.02388
T3	0.01666
T4	0.02815
R1	0.02046
R2	0.02010
R3	0.01709
R4	0.01565
X1	0.01676
X2	0.02240
X3	0.02116
X4	0.02157
X5-1	0.02647
X5-2	0.02592
X5-3	0.02460
X5-4	0.02565
X5-5	0.02427
CT	0.01398

Among those variances, the *p*-value of the model was only 0.0033, so the model can be considered as reliable. The *p*-values of *D*, *DH* and H^2 were less than 0.005, suggesting a very significant influence on the average COFs. The *p*-values of *H* and D^2 were both less than 0.05, indicating a significant impact on the target COFs as well. The determination coefficient (R^2) and the correction determination coefficient (R^2_{Adj}) were both above 0.8, while the difference between the predicted correction coefficient (R^2_{Pre}) and the correction determination coefficient (R^2_{Adj}) exceeded 0.2, implying a significant error between the predicted and actual results of the model. Although the prediction accuracy of the model was not high, the signal-to-noise ratio (*PA*) was still greater than 4, confirming that the model was reliable enough for parameter optimization [37].

Table S2. Analysis of the BBD-RSM model.

Source	Sum of Squares	Mean Square	F-value	p-value	Significance
Model	2.210E-004	2.455E-005	9.81	0.0033	*****
D	5.235E-005	5.235E-005	20.91	0.0026	*****
P	2.546E-006	2.546E-006	1.02	0.3468	x
H	2.312E-005	2.312E-005	9.23	0.0189	**
DP	1.625E-007	1.625E-007	0.065	0.8062	x
DH	4.180E-005	4.180E-005	16.70	0.0047	*****
PH	6.843E-006	6.843E-006	2.73	0.1423	x
D^2	1.763E-005	1.763E-005	7.04	0.0328	**
P^2	3.899E-006	3.899E-006	1.56	0.2522	x
H^2	6.559E-005	6.559E-005	26.20	0.0014	*****
Residual	1.753E-005	2.504E-006			
Lack of Fit	1.414E-005	4.712E-006	5.56	0.0655	x
Pure Error	3.391E-006	8.478E-007			
Cor Total	2.385E-004				
R^2=0.9265	R^2_{Adj}=0.832	R^2_{Pre}=0.0295	PA=9.543		
Notes: ***** very significant (p<0.005); ** significant (p<0.1); * not significant (p>0.1);					

The obtained regression model for predicting the average COF is as follows:

$$\mu = -0.059052 + 4.44653\times10^{-4} D + 1.5672\times10^{-3} P + 3.30922\times10^{-3} H + 9.71325\times10^{-7} DP - 6.46531\times10^{-6} DH - 3.15162\times10^{-5} PH - 8.1855\times10^{-7} D^2 - 5.58769\times10^{-5} P^2 - 3.94696\times10^{-5} H^2 \quad (1)$$

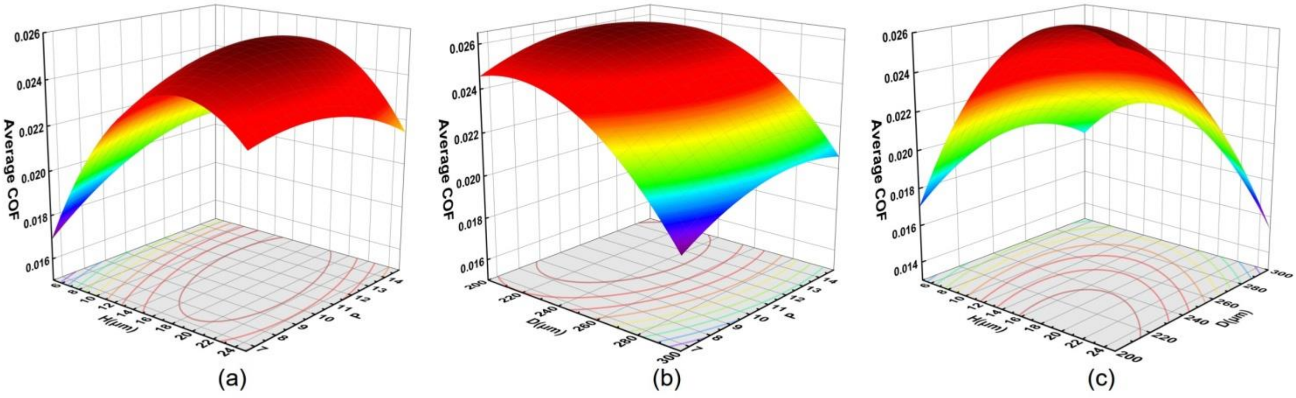

Figure S1: Response surfaces of the interaction among three factors (D, P, H) on the average COFs of the PEEK-1040 steel friction-pairs. (a) Response surface between P and H; (b) Response surface between D and P; (c) Response surface between D and H.

As the average COF was the dependent variable and the other horizontal axes were the independent variables, the response surfaces between the average COF and the three dimple parameters (D, P, H) are exhibited in Figure 9. When one horizontal axis is fixed, the shape of the response surface shows a downward opening parabola along the vertical axis (the other horizontal axis is the independent variable). When the influence of this horizontal axis on the average COF was significant, the opening of the parabolic became narrower, further making the entire response surface sharper. Therefore, based on the sharpness of the response surfaces, the influence of the dimple

parameters on the average COF is in a order of: *D*>*H*>*P*, which is aligned with the *p*-values of *D*, *H* and *P* and also consistent with previous discussions. To keep the same AASDs, i.e., 1.5°/2.5°, the dimple parameters were chosen within the three levels set: *D*=208.32 μm, *P*=6.97%, and *H*=5.07 μm, with a target average COF of 0.015 and a TEVD of 0.60. After comprehensively considering the performance of the laser marking machine and the AASDs, the parameters were finally rounded to *D*=200 μm, *P*=6.6%, and *H*=5 μm, with an adjusted target average COF of 0.014 and an adjusted TEVD of 0.54.

2 Experimental validation

The textured 1040 steel discs with rounded dimple-parameters were tested against PEEK rings three times to access the tribological performance after BBD-RSM optimization under the identical conditions. Note that: A brand new 1040 steel disc was used in each test. The group with the lowest COF curve among the 17 groups was named as LT (i.e., R4). The dimple-textured group with optimized parameters was coded as OT. Their COF curves were compared in Figure 2(a) alongside the COF curve for the smooth reference (CT).

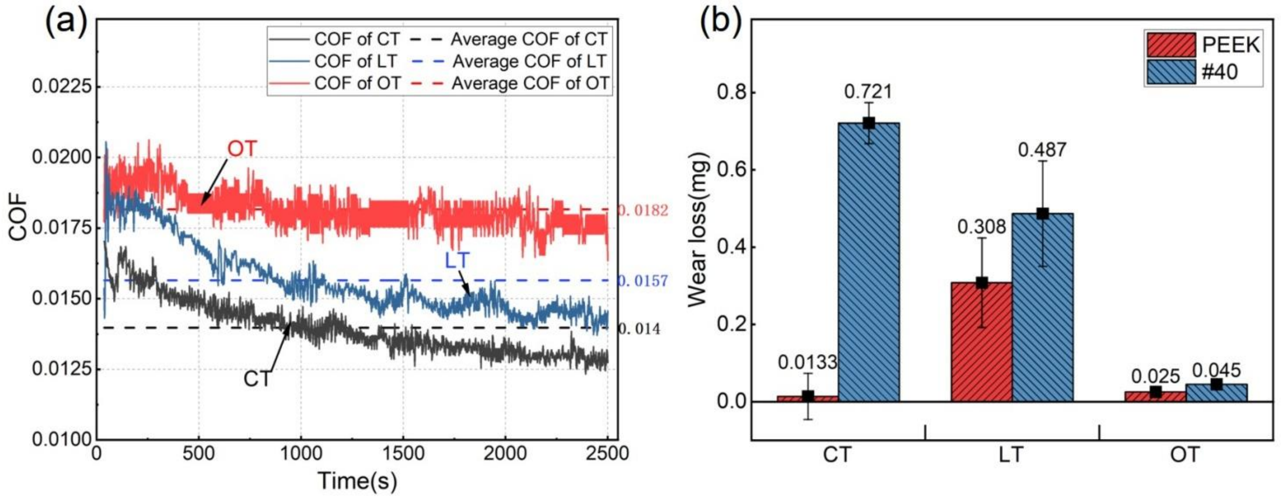

Figure S2: The COF curves and wear losses of three groups (OT, LT and CT). (a) COF curves of OT, LT and CT; (b) wear losses of OT, LT, and CT.

Each COF curve in Figure 2(a) presented the average value of three repeated tests at each recording time-point. The average COFs of OT, LT and CT groups were 0.018, 0.016 and 0.014, respectively, consistent with their COF curves. The COF curve of CT group was still the lowest, while the curve of OT group was the highest. Additionally, the COF curve of OT group exhibited greater fluctuations throughout the test process compared to LT and CT groups. So, micro-dimples do not effectively reduce the average COFs or COF fluctuations of the PEEK-1040 steel friction-pairs under full film lubrication, within the parameter ranges set. The final average COF (0.018) of OT group was 0.004 higher than the predicted average COF (0.014) by response surface method, with an error of 21.4%. The accuracy of the BBD-RSM model was clearly unsatisfactory.

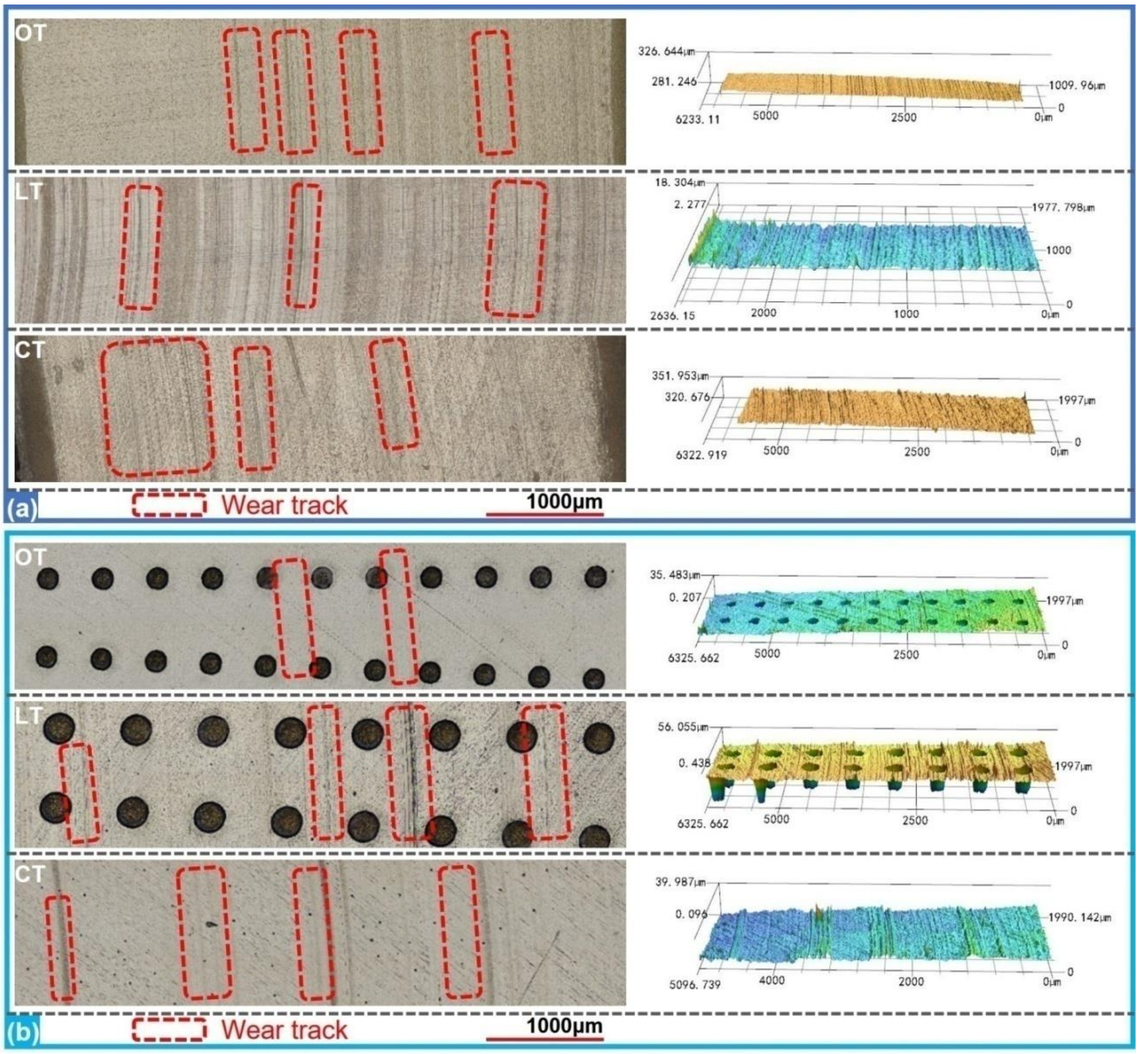

Figure S3: Representative worn surfaces and morphologies of the contact-surfaces of the OT, LT and CT groups after ultrasonic cleaning. (a) PEEK rings; (b) 1040 steel discs.

The mass losses of OT, LT and CT groups are compared in Figure 2(b). Regarding the 1040 steel discs, the wear loss of CT group was the highest, followed by LT group. The mass losses of both the PEEK ring and the 1040 steel disc in OT group were significantly reduced. Therefore, although the dimple parameters after optimization cannot reduce the average COFs of PEEK-1040 steel friction-pairs, they can significantly enhance their wear resistance. Compared to LT group, the wear losses of the PEEK ring and 1040 steel disc in OT group were decreased by 90.4% and 93.1%, respectively. Compared with the results of CT group, the wear loss of the 1040 steel disc in OT group was also reduced by 93.8%.

The representative worn surface of the PEEK ring in OT group was quite smooth. Only very few visible wear marks on the contact surface (see Figure 3(a)). This was in sharp contrast to the worn surfaces on the PEEK rings in LT and CT groups. The embedded metal debris was also unwatchable. As for the 1040 steel discs, only a few slight wear marks were observed on the 1040 steel disc in OT group (see Figure 3(b)). The PEEK transfer film left on the contact-surface was also very few, which could be confirmed by its 3D morphology.

3 Orthogonal design method

A full experiment provides a clear analysis of the relationships between factors and indicators, but the number of trials can be overwhelming—especially when there are many factors and each factor has several levels. For example, in a three-factor, three-level experiment, a full factorial experiment would require $3^3 = 27$ combinations, not including the repeated tests for each combination.

Orthogonal design method (ODM) is a highly efficient, rapid, and cost-effective experimental design method. It uses orthogonal tables to select a representative set of "uniformly distributed and comparable" points from a comprehensive experiment. So, this allows for significantly fewer trials while still providing a clear explanation of the relationship between experimental conditions and performance indicators. So, if we use an L9(3^4) orthogonal table to design a three-factor, three-level experiment, only 9 tests are needed; even if we use an L15(3^7) orthogonal table, 15 trials are required only. This clearly reduces the workload.

Orthogonal tables have two key properties:

(1) The frequency of each number appearing in a column is the same.

(2) The frequency of each pair of numbers appearing in any two columns is also the same.

Because of these two properties, when used to design experiments, the combinations of factor levels are balanced.

Orthogonal tables are commonly denoted by an "L," with the following common forms: L8(2^7), L9(3^4), L16(4^5), L8(4×2^4), L12(2^11), etc. The numbers in the notation indicate the following:

L8(2^7): "7" is the number of columns (maximum number of factors), "2" is the number of levels for each factor, and "8" is the number of rows (the number of trials).

L16(2×3^7): This table has 7 columns with 3 levels, and 1 column with 2 levels. It indicates that with 16 trials, we can study one 2-level factor and seven 3-level factors.

In an orthogonal table with m rows and n columns (where m and n are positive integers), the number of trials (rows) is calculated as:

Number of trials = Σ (number of factors × (level of each factor - 1)) + 1.

For example, for L8(2^7), we have: 8 = 7 × (2 - 1) + 1.

By using this formula, you can determine the minimum number of trials required based on the factor levels and select an appropriate orthogonal table. For example, if you want to examine five 3-level factors and one 2-level factor, the minimum number of trials would be: 5×(3-1)+1×(2-1)+1=12 trials. Therefore, you would choose a table with at least 13 rows that includes both 2-level and 3-level columns, such as L16(2×3^7).

If six factors are chosen, each with five levels, a full factorial experiment would require 5^6 = 15,625 trials, which is practically impossible. However, using the orthogonal experimental method,

only 25 trials are needed. In a sense, these 25 trials represent the 15,625 trials of a full experiment.

The purpose of post-experiment data analysis is to identify the optimal factors and the best experimental combination. There are two main analysis methods: range analysis and variance analysis. Range analysis, also known as intuitive analysis, is simple, visual, and easy to understand, making it the most commonly used method for analyzing orthogonal experimental results.
